# Dental pulp stem cells‐based therapy for the oviduct injury via immunomodulation and angiogenesis in vivo

**DOI:** 10.1111/cpr.13293

**Published:** 2022-07-12

**Authors:** Lihua Luo, Zhenjie Xing, Xiangyan Liao, Yejian Li, Yu Luo, Yilong Ai, Yan He, Qingsong Ye

**Affiliations:** ^1^ School and Hospital of Stomatology Wenzhou Medical University Wenzhou China; ^2^ Center of Regenerative Medicine Renmin Hospital of Wuhan University Wuhan China; ^3^ Tianyou Hospital Wuhan University of Science and Technology Wuhan China; ^4^ Foshan Stomatological Hospital, School of Medicine Foshan University Foshan China

## Abstract

**Objectives:**

As a result of the current limitation of therapeutic strategies, the repair and regeneration of oviduct injuries required an alternative treatment. We present a novel approach to treat oviduct injuries through a dental pulp stem cells (DPSCs)‐based therapy.

**Materials and Methods:**

In vitro and in vivo models have been established. Immunofluorescence staining, flow cytometry and enzyme‐linked immunosorbent assay (ELISA) analysis were used to investigate the features and angiogenic properties of DPSCs, as well as their impact on macrophages, in vitro. For the in vivo experiment with female SD rat model, immunohistochemical staining and ELISA analysis were used to assess the effects of DPSCs on the repair and regeneration of damaged oviducts.

**Results:**

The present data showed that intraperitoneal injection of DPSCs reduced the expression of IL‐6 and TNF‐α to inhibit the immunoreaction in injured sites, as well as increased the expression of VEGF to promote the in situ formation of vessel‐like structures, thus the repair and recovery process could be initiated.

**Conclusions:**

We concluded that DPSCs‐based therapy could be a novel potential technique for restoring the structure and function of damaged oviduct by enhancing immuno‐regulated effect and promoting angiogenic property.

## Graphical abstract

Dental pulp stem cells (DPSCs) were obtained from the pulp tissues which were extracted from the impacted third molars. The suspension of DPSCs was used to restore and treat the injured oviduct by intraperitoneal injection.

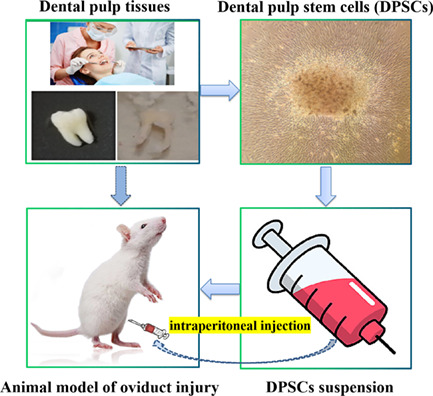

## 1 INTRODUCTION

The oviduct, as an important organ of gestation, was not only responsible for the transport of sperm and eggs but also played a key role in the nutritional function of embryos and so on. According to recent reports, oviduct injuries were the leading cause of female infertility, accounting for 30%–35% of cases.^1^ Furthermore, the cilia of the oviduct would flow towards the direction of the uterus, which was an important factor in the transport of the fertilized egg. In addition, the morphological damage of cilia would increase the incidence of infertility and ectopic pregnancies.^2^ As a result, reconstruction of the structure and function of the oviduct cilia was a key component in increasing the pregnancy rate.

Many recent studies indicated that mesenchymal stem cells (MSCs) had therapeutic effects on the repair and regeneration of the injured oviduct.^3^ MSCs were considered a promising treatment strategy for regenerative medicine for the following reasons. First, MSCs were capable of self‐renewal and multipotential differentiation,^4^ which could be induced to differentiate into vascular endothelial cells,^5^ nerve cells,^6^ and smooth muscle cells,^7^ which would provide the replacement cells in injured sites. Second, during the repair and regeneration of oviduct injuries, MSCs demonstrated the ability to produce a large number of soluble small molecules that induced the creation of vessel‐like structures.^8^ Finally, MSCs also possessed immunoregulatory properties as well as had the capability to modulate the inflammatory cells, which could help with immunoreaction and reconstitution of normal tissue in damaged sites.^9^, ^10^

Dental pulp stem cells (DPSCs), as one member of MSCs, had the advantages of being convenient sources and could replicate in large quantities in a short time.^11^ Furthermore, DPSCs could be isolated from the impacted third molars and orthodontically extracted premolars, avoiding ethical issues and invasive damage to the body.^12^ DPSCs also possessed MSCs‐like properties such as multi‐differentiation and immunomodulatory effect. Some studies have recently shown that DPSCs can not only secrete beneficial angiogenic factors but can also differentiate into vascular endothelial cells to promote new angiogenesis.^13^, ^14^, ^15^ Furthermore, DPSCs have been shown to possess anti‐inflammatory properties, which can promote the transformation of macrophages into anti‐inflammatory phenotypes and reduce the level of inflammatory factors.^16^, ^17^, ^18^ As a result of their angiogenic and anti‐inflammatory properties, DPSCs were considered an effective therapeutic pathway for tissue regeneration. Furthermore, DPSCs have been used for the treatment of injurious diseases in animal models, such as liver fibrosis^19^ and myocardial infarction.^20^ However, no studies have been done to prove that DPSCs‐based stem cell therapy could be used for the repair and regeneration of oviduct injuries. To investigate the effect of DPSCs on the treatment of oviduct injuries, we transplanted DPSCs suspension into the animal model by intraperitoneal injection.

## 2 MATERIALS AND METHODS

### 2.1 Isolation and culture of DPSCs

The DPSCs were collected and treated as previously described.^21^ Dental pulp tissues were extracted briefly from the impacted third molars and digested with collagenase type I (Gibco, USA) and dispase (Sigma, Germany) for 30 min in the incubator. The cellular suspension was then incubated with α‐modified Eagle's medium (α‐MEM, Gibco, USA) supplemented with 20% fetal bovine serum (FBS, Gibco, USA), and 1% penicillin–streptomycin solution (Gibco, USA) in 5% CO_2_ at 37°C. The culture medium was replaced every 3 days.

### 2.2 Identification of DPSCs

The mesenchymal phenotype of DPSCs was assessed by immunofluorescence staining. At 37°C, DPSCs were fixed with 4% formaldehyde for 15 min and were permeabilized for 10 min with 0.1% Triton X‐100. After blocking with 5% goat serum, the DPSCs were incubated with the primary antibodies of CD146 and CD44 (Proteintech, USA) at 4°C overnight. DPSCs were then stained by the goat anti‐rabbit IgG secondary antibody (Abcom, England). The images were analysed by fluorescence microscopy (Eclipse 80i, Nikon, Japan). Flow cytometry was also used to detect the stemness of DPSCs using the primary antibodies of human CD73, CD105 and CD34 (Biolegend, USA). The data was then analysed using the flow cytometer (BD FACS Verse, USA).

### 2.3 Co‐culture of DPSCs and RAW264.7 cells in vitro

For co‐culture experiments, 700 μl of RAW264.7 cells suspension (2 × 10^5^ cells/ml) and 400 μl of DPSCs suspension (2 × 10^4^ cells/ml) were plated onto 24‐well plates and 0.4‐μm pore size inserts (Millipore, USA) for pre‐culture, respectively. Meanwhile, the RAW264.7 cells were treated with lipopolysaccharide (LPS, 10 ng/ml). After 24 h, the inserts were transferred into the 24‐well plates for co‐culture for another 24 h. Then, the mediums were collected for ELISA analysis, and the cells were used for the following test.

Immunofluorescence staining was performed to assess the expression level of IL‐6. RAW264.7 cells were fixed with 4% formaldehyde and blocked with 5% goat serum containing 0.1% Triton X‐100, before being incubated with the primary antibody of IL‐6 (Abcam, England) at 4°C overnight, followed by the appropriate horseradish peroxidase‐conjugated secondary antibody for another 1 h. Photographs were imaged by a fluorescence microscope (Eclipse 80i, Nikon, Japan).

### 2.4 The angiogenic potentiality of DPSCs

GelMA hydrogel precursor was synthesized by reacting gelatin with methacrylic anhydride according to our previously established protocols.^22^ In this study, the 10% (w/v) GelMA hydrogel was used for the scaffold to 3D encapsulate DPSCs with a density of 6 × 10^5^ cells/ml. Briefly, DPSCs were collected and re‐suspended in GelMA solutions. Then the 50 μl mixture was added to each well (96‐well plate) and photo‐cross‐linked to obtain GelMA hydrogel containing DPSCs. Finally, the hydrogel samples were cultured with endothelial cell growth medium‐2 (EGM‐2™, Lonza, Switzerland) in 5% CO_2_ at 37°C. The medium was replaced every 3 days. The cells were detected via immunofluorescence staining with β‐tubulin III (Cell Signalling Technology, USA) after being induced with EGM‐2™ for 7 days.

### 2.5 Establishment of the animal model

Sprague–Dawley female rats (180–200 g, Animal Center of Chinese Academy of Science, Shanghai, China) were acclimatized in the animal care facility before their weight was more than 300 g. The experimental animals were randomly divided into three groups, including the control group (*N* = 6), the model group (*N* = 6), and the DPSCs group (*N* = 6). All animals were anaesthetised by the intraperitoneal injection of 2% pentobarbital sodium at a dose of 3 ml/kg. After anaesthesia, back hair was shaved and the skin was sanitized with 70% alcohol solution. To expose the uterus and oviducts, skin incisions extended along the middle of the back and muscles were split, and 0.1 ml of anhydrous ethanol was injected into the right oviduct of each rat. The muscle and skin of the rats were then sutured in layers. After 2 h, 1 ml DPSCs suspension (1 × 10^6^ cells/ml) was transferred into rats by intraperitoneal injection in the DPSCs group. Meanwhile, over the next 3 weeks, the DPSCs suspension would be injected once a week. As for the model group, the saline solution was injected into the rats after surgery. Furthermore, the rats in the control group were unharmed. The oviduct tissues and blood were collected after 4 weeks for further analyses.

### 2.6 Detection of serum IL‐6 and TNF‐α expression

The rat's blood was immediately centrifuged at speed of 1500 g for 10 min at 4°C after being drawn from the heart. Following that, the serum of blood was collected to measure the concentrations of IL‐6 and TNF‐α using ELISA kits (Takara, Japan) according to the manufacturer's protocols.

### 2.7 Histologic evaluation

The sections (5 μm thickness, paraffin‐embedded) were stained by Masson trichrome staining reagent and haematoxylin–eosin (Keygen, Nanjing, China) according to the manufacturer's instructions for HE and Masson trichrome staining. Images were obtained by a light microscope (TS100, Nikon, Japan).

### 2.8 Immunohistochemical (IHC) analysis

The oviduct sections were deparaffinized and rehydrated by gradient elution using xylene and ethanol for IHC analysis. The sections were then incubated with the primary antibodies of vascular endothelial growth factor (VEGF) (Abcam, England), IL‐6 (Affinity, USA) and TNF‐α (Proteintech, USA) at 4°C overnight. After washing with PBS, the sections were incubated with the goat anti‐rabbit IgG secondary antibody containing horseradish peroxidase (HRP)‐streptavidin reagent (Abcam, England). Finally, the images were observed by a light microscope (TS100, Nikon, Japan).

### 2.9 Statistical analysis

All the data were presented in mean ± standard deviation. Statistical differences were performed by one‐way analysis of variance and Student's *t*‐test. *p* < 0.05 was considered significantly statistical. Statistical analyses were calculated by SPSS 19.0 (SPSS, Chicago, IL).

## 3 RESULTS

### 3.1 The culture and identification of DPSCs

**FIGURE 1:**
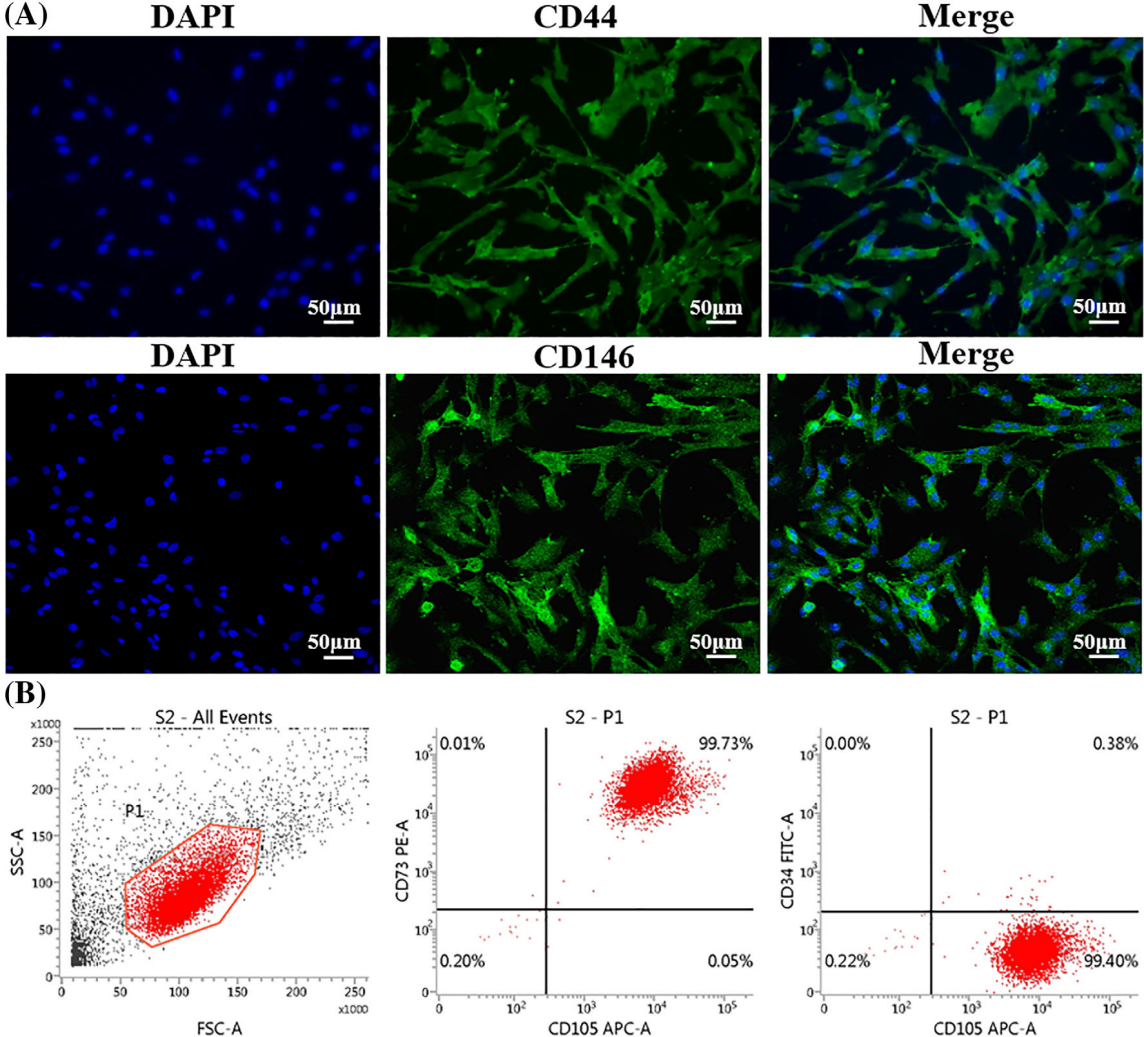
The identification of DPSCs. (A) The expression of CD44 and CD146 was evaluated by immunofluorescence staining. (B) The expression of CD105, CD73 and CD34 was evaluated by flow cytometry

As shown in the immunofluorescence staining results (Figure 1A), DPSCs positively expressed the MSCs‐like phenotypic markers such as CD44 and CD146. Meanwhile, the flow cytometry data indicated that DPSCs had a positive CD105 and CD73 expression but negative CD34 expression (Figure 1B).

### 3.2 The expression of IL‐6 in vitro cell experiments

**FIGURE 2:**
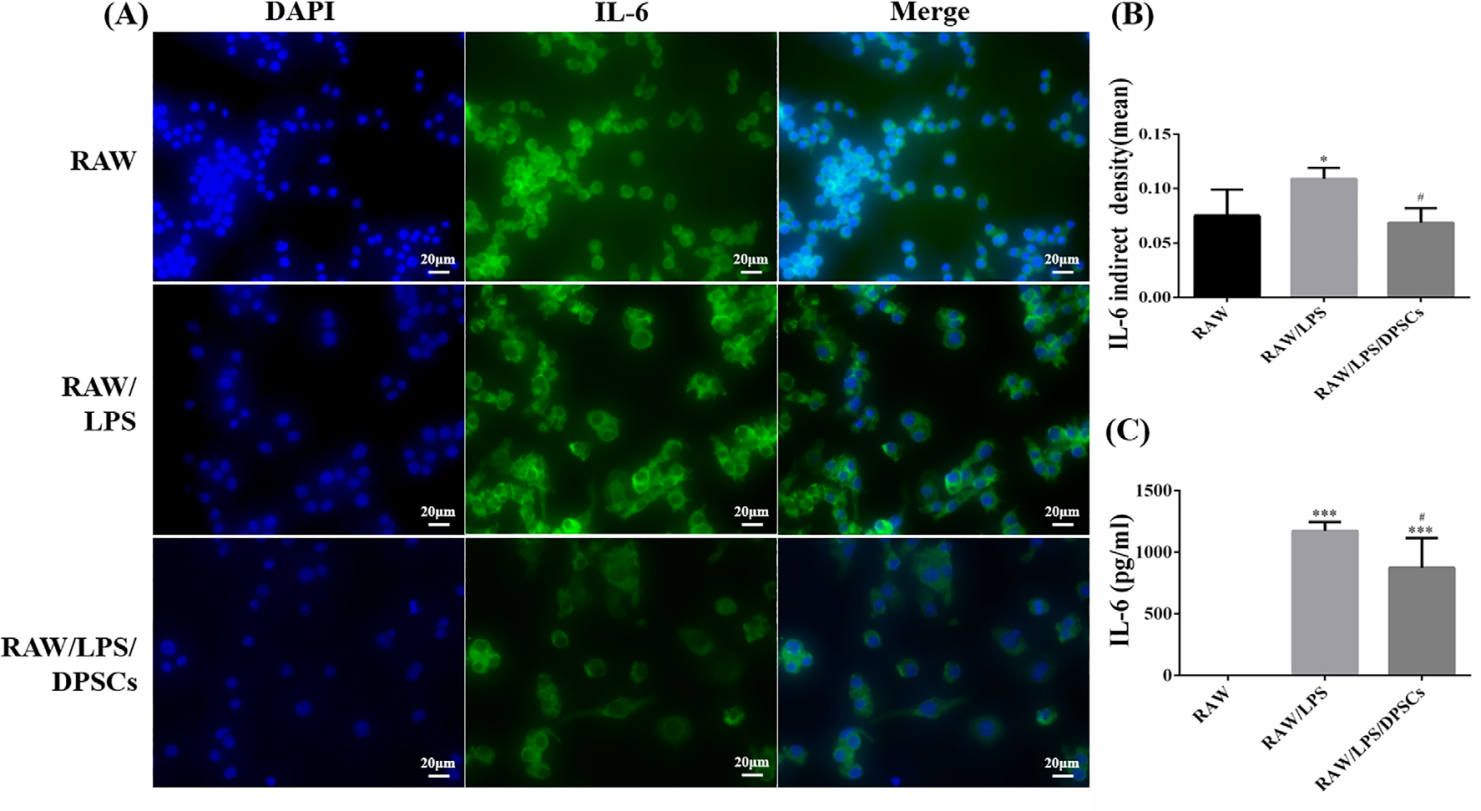
Detection of IL‐6 expression in vitro experiments. (A) Immunofluorescence staining of IL‐6 in RWA264.7 cells by co‐cultured with DPSCs. (B) Quantitative analysis of IL‐6 intensity. *p < 0.05 versus the RAW group, ^#^ *p* < 0.05 versus the RAW/LPS group. (C) The analysis of IL‐6 contents in the cultured mediums. ***p < 0.001 versus the RAW group, ^#^ *p* < 0.05 versus the RAW/LPS group

As shown in Figure 2A, the results suggested that there was a positive expression of IL‐6 in the RAW group. The expression of IL‐6 increased in the RAW/LPS group after being treated with LPS. Meanwhile, the RWA264.7 cells became larger and presented a polar state in the RAW/LPS group. However, after co‐cultured with DPSCs for 24 h, the expression of IL‐6 decreased in the RAW/LPS/DPSCs group. Furthermore, there was a statistical difference in IL‐6 expression levels between the RAW group and the RAW/LPS group as well as the RAW/LPS group and the RAW/LPS/DPSCs group (Figure 2B, *p < 0.05). Furthermore, Figure 2C indicated that the concentration of IL‐6 in cultured mediums was the highest in the RWA/LPS group. After co‐cultured with DPSCs, the secretion of IL‐6 was decreased and existed a statistical difference compared to the RAW/LPS group (^#^ *p* < 0.05).

### 3.3 The angiogenic property of DPSCs

**FIGURE 3:**
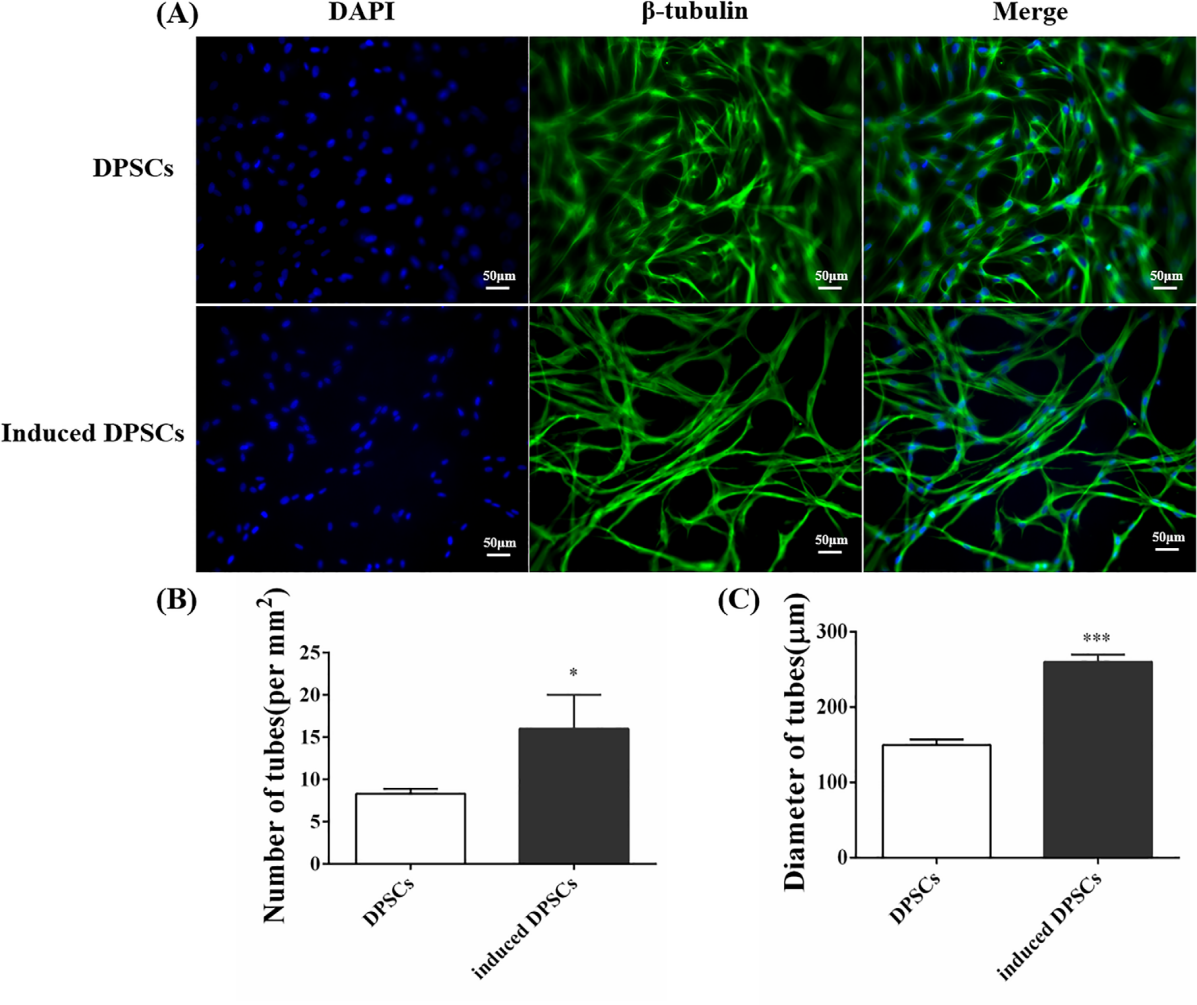
The angiogenic property of DPSCs by directly induced with EGM‐2™ for 7 days. (A) Immunofluorescence staining of β‐tubullin III. (B) The number of the formed tubes. *p < 0.05 versus the DPSCs group. (C) The diameter of the formed tubes. ***p < 0.001 versus the DPSCs group

As shown in Figure 3, the results illustrated that DPSCs which were induced by EGM‐2™ exhibited a promising angiogenic property. In the morphology, the induced‐DPSCs gradually elongated and extended many branches, which cross‐linked to form the network structures (Figure 3A). Furthermore, the number and diameter of vessel‐like structures in the induced‐DPSCs group were considerably higher than that in the DPSCs group, with statistical significance (Figure 3B,C, *p < 0.05, ***p < 0.001).

### 3.4 HE and Masson trichrome staining

**FIGURE 4:**
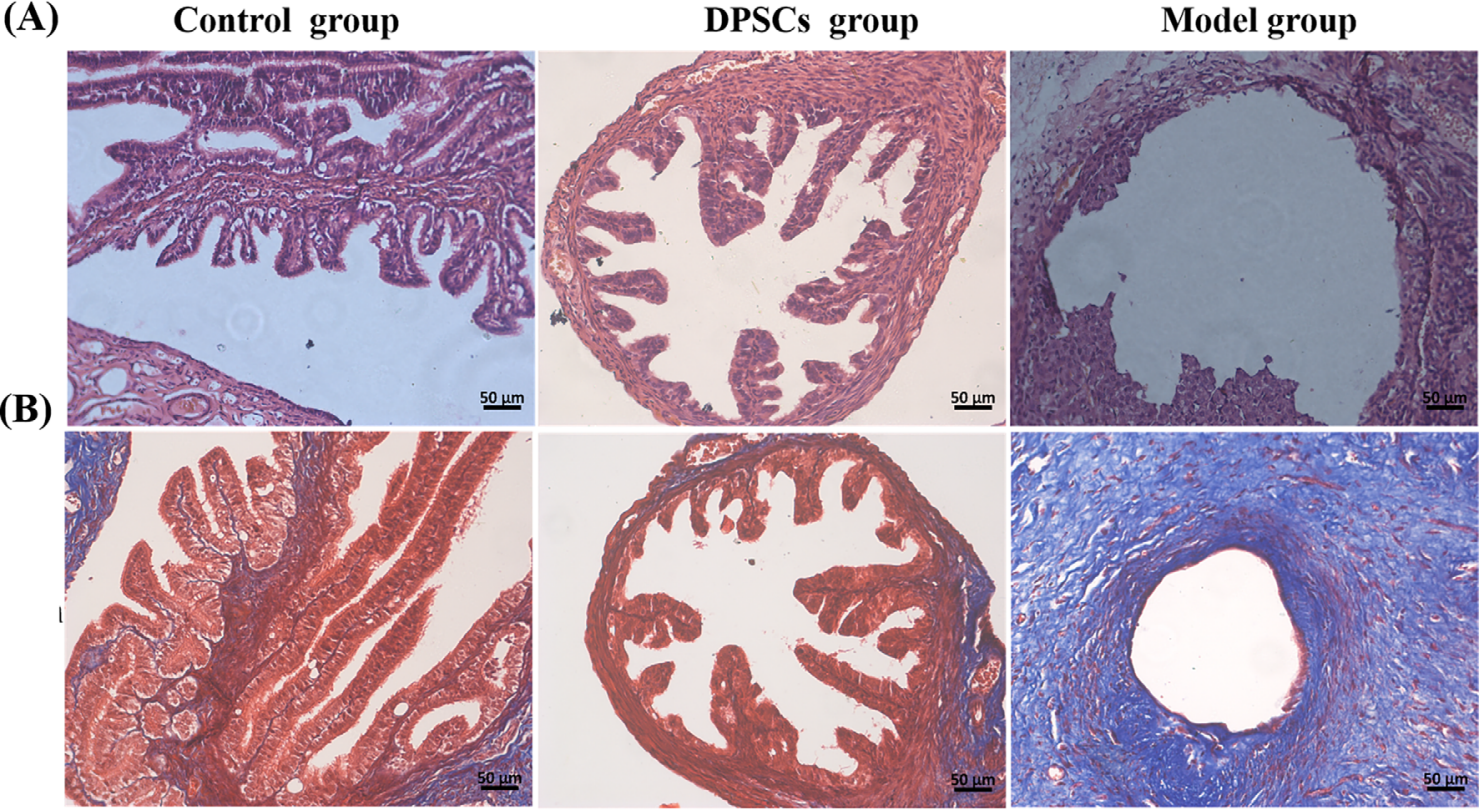
Histologic evaluation of oviduct tissues after 30 days post‐surgery. (A) Haematoxylin–eosin (HE) staining. (B) Masson trichrome staining

Thirty days post‐surgery, the HE results demonstrated that the mucosal membrane protruded into the lumen and formed the mucosal folds with secondary branches, and the epithelial cells were orderly arranged in folds in the control group. In the model group, the mucosal folds disappeared and the epithelial structures were destroyed. In contrast, in the DPSCs group, epithelial folds were significantly increased and secondary branches were visible (Figure 4A). For the Masson trichrome staining, normal submucosal collagen fibres were stained with blue, and cytoplasm, muscle, and cellulose were shown in red. In the control group, the folds of mucosa were intact, and the submucosa was composed of stromal cells and loose collagen fibres. In the model group, the epithelial structures were damaged, and the deposition of blue collagen fibres in the submucosa was increased, which had been shown corrugated cords and irregular arrangement. After being treated with DPSCs, the structures of mucosal epithelium recovered, and little collagen fibres were interspersed with stromal cells (Figure 4B).

### 3.5 Immunohistochemical assay

**FIGURE 5:**
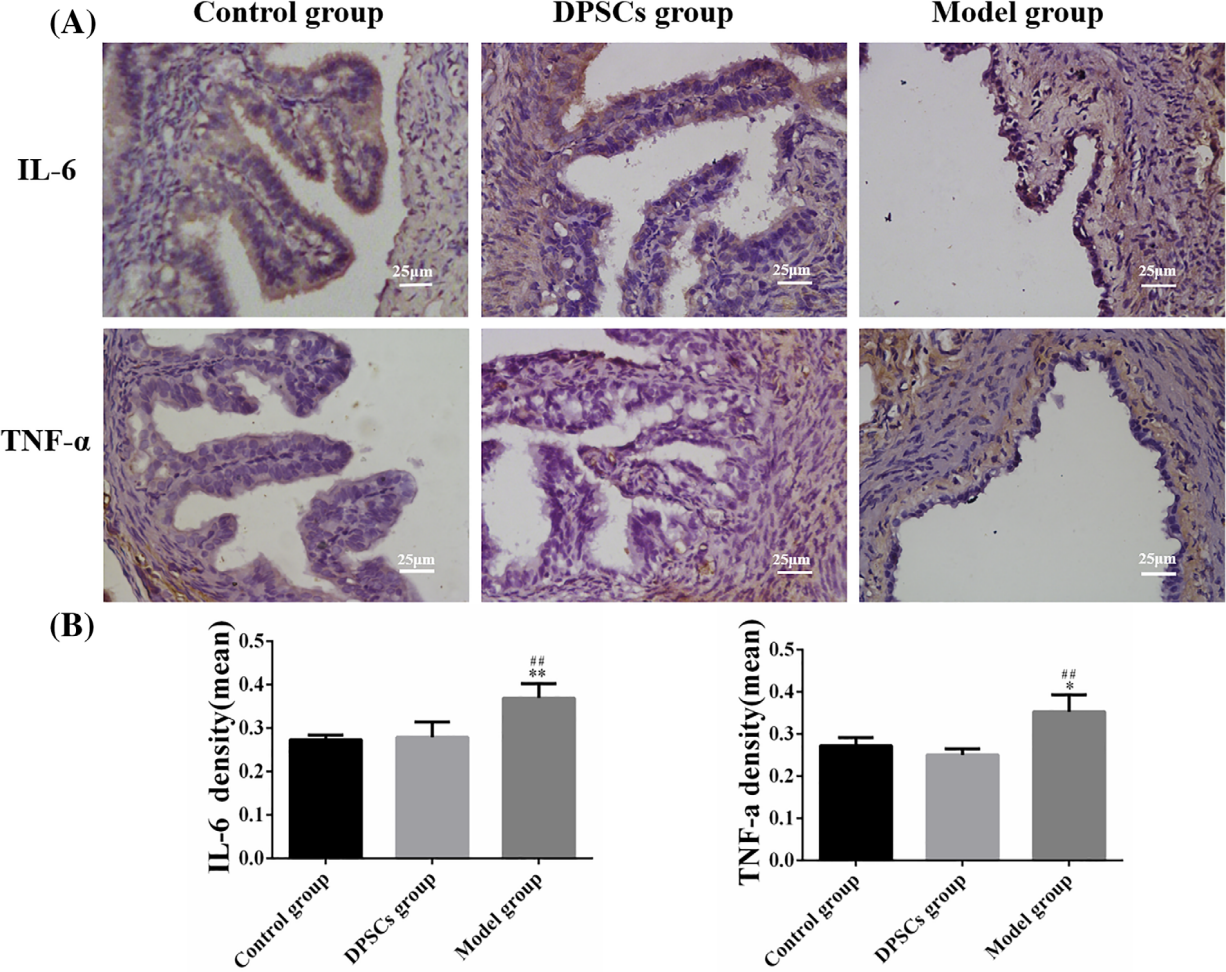
Analyses the expression of IL‐6 and TNF‐α in oviduct tissues. (A) The expression levels of IL‐6 and TNF‐α in oviduct tissues on the 30 days after animal experiments. (B) Quantitative analysis of IL‐6 and TNF‐α positive cells. The quantification results were obtained by Image pro plus. Data were presented as mean ± standard error from 3 rats in each group. *p < 0.05, **p < 0.01 versus the control group; ^##^ *p* < 0.01 versus the DPSCs group

The expression levels of IL‐6 and TNF‐α in the fallopian tubes of the different experimental groups were presented in Figure 5. The expression of IL‐6 and TNF‐α were strong in the model group. There was a weak expression of IL‐6 and TNF‐α presented in the DPSCs group, which was similar to that in the control group (Figure 5A). Furthermore, the statistical data suggested that the expression levels of IL‐6 and TNF‐α in the model group were higher than that in the control group as well as in the DPSCs group. Furthermore, there was no significant difference between the DPSCs group and the control group (Figure 5B).

**FIGURE 6:**
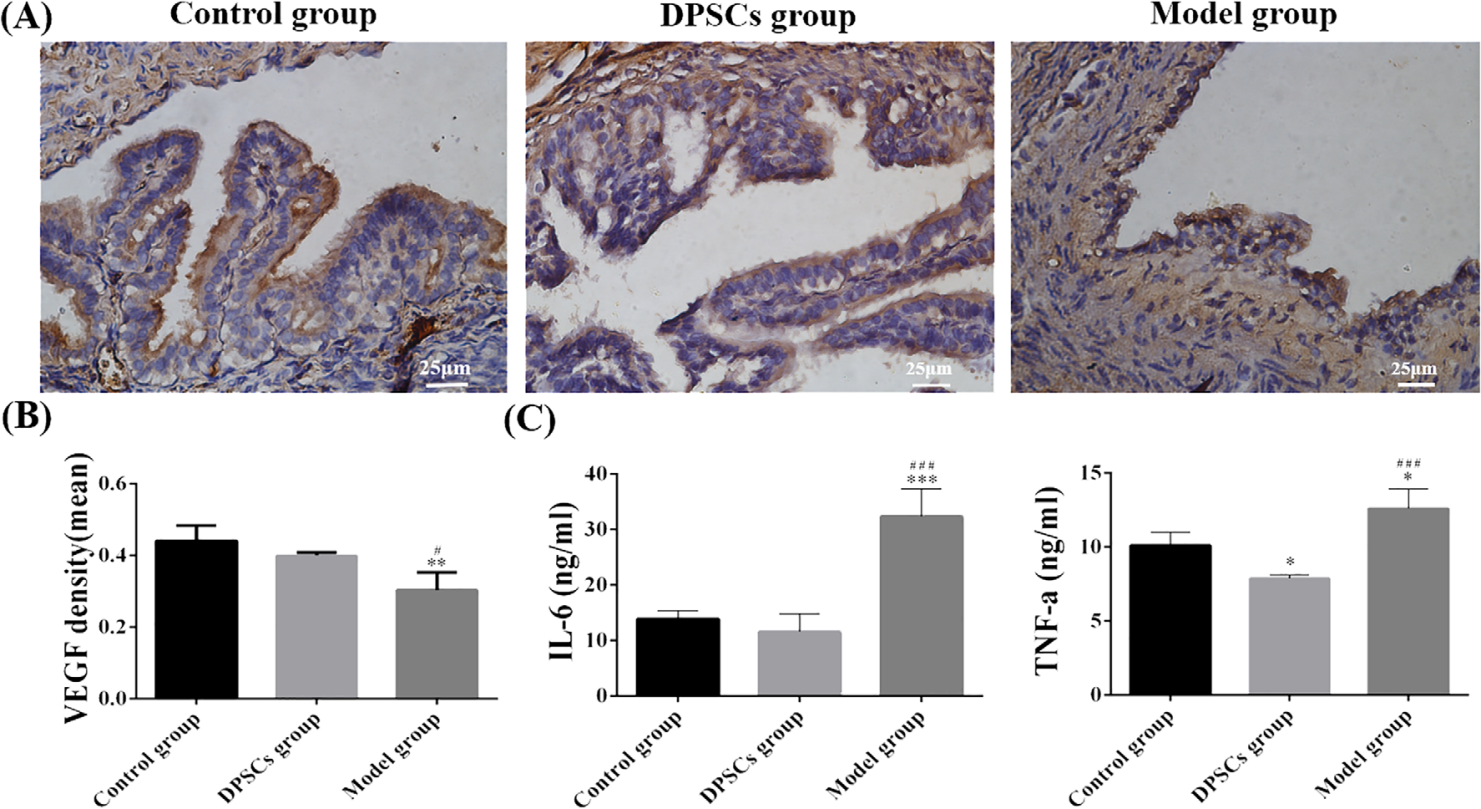
Detection of VEGF expression in oviduct tissues and analysis of IL‐6 and TNF‐α expressions in serum. (A) The expression of VEGF in oviduct tissues on the 30 days after animal experiments. (B) Quantitative analysis of VEGF positive cells. (C) The ELISA analyses of IL‐6 and TNF‐α contents in serum after 30 days post‐surgery. Data were presented as mean ± standard error from 3 rats in each group. The quantification results were obtained by Image pro plus. *p < 0.05, ***p < 0.01 versus the control group; ^###^ *p* < 0.001 versus the DPSCs group

In terms of VEGF staining, the expression of VEGF in the DPSCs group was increased compared to the model group's modest expression (Figure 6A). Meanwhile, there was a significant difference between the model group and the DPSCs group, whereas no significant statistical difference between the DPSCs group and the control group (Figure 6B).

### 3.6 The expression levels of serum IL‐6 and TNF‐α

The results indicated that the expression levels of serum IL‐6 and TNF‐α in the model group were obviously higher than that in the control group, as shown in Figure 6C, and the difference existed significantly statistically. The concentration of serum IL‐6 decreased after treatment with DPSCs and returned to normal levels. Furthermore, there was no statistical difference between the control group and the DPSCs group. Similarly, the concentration of serum TNF‐α in the DPSCs group was not only far lower than that in the model group but even lower than that in the control group. There were also statistically significant differences between the experimental groups.

## 4 DISCUSSION

Ovarian duct injuries can be caused by a variety of factors, including infection, ectopic pregnancy, tubal surgery, endometriosis and chemotherapy exposure could be caused oviduct injuries.^23^ However, there was no ideal approach that could restore the oviduct tissues structure and function without any sequela. MSCs possessed great proliferation and excellent multipotency which could be induced to differentiate into mature specialized cells. In addition, MSCs had the ability to modulate the immunoreaction and inflammation. Moreover, MSCs were able to secrete the extracellular vesicles such as exosomes, apoptotic bodies, nanoparticles and microvesicles, which provided great paracrine power and significant immunomodulation for the repair and regeneration of injured tissues.^24^ Therefore, tissue repair and regeneration have been shown to benefit from stem cell biotherapy. DPSCs were one kind of MSCs, derived from the euangiotic dental pulp, and had huge prospects in tissue regeneration.^11^ DPSCs positively expressed MSCs‐like markers such as CD146 and CD44, as well as CD 105 and CD73, according to our findings. Furthermore, both in vitro and in vivo, DPSCs could suppress the expression of anti‐inflammatory factors such as IL‐6 and TNF‐α. In addition, DPSCs had the angiogenesis ability to promote the formation of vessel‐like structures. As a result, these capabilities of DPSCs could provide a promising strategy for the treatment of oviduct injuries.

The normal structure of the oviduct consisted of the submucosal muscle layer and mucosal folds. Single columnar epithelium and cilia created the oviduct mucosa folds. Furthermore, embryo transport needed the movement of cilia and coordination of cellular secretions.^25^ As a result, the function of the oviduct was determined to some extent by the morphology and function of mucosal folds. As shown in Figure 4, the HE and Masson trichrome staining results illustrated that the structures of mucosal folds had disappeared in the model group. Meanwhile, there were lots of collagen fibres immersed in the muscle layer. The length and shape of mucosal folds in the DPSCs group were significantly restored in the DPSCs group after treatment and were nearly identical to those in the control group. As for the pro‐inflammatory cytokines, TNF‐α and IL‐6 were closely related to reproductive immunization.^26^, ^27^ Both of them could increase the vascular permeability and promote apoptosis of the trophoblastic cells.^28^ Some studies suggested that DPSCs could regulate inflammatory factors such as IL‐6 and TNF‐α to repair and regenerate the damaged tissues.^29^ As our previous studies described, DPSCs could inhibit the release of IL‐6.^21^ In this research, the results of ELISA and IHC assays had been shown that the secretion of IL‐6 and TNF‐α was decreased after being treated with DPSCs, and there was no significant statistical difference between the control group and the DPSCs group (Figures 5 and 6C). The results were consistent with the conclusion of other researchers. According to Omi et al,^18^ DPSCs could suppress the inflammation responses to promote the repair of sciatic nerves in injured sites. Makino et al^30^ demonstrated that the conditioned medium of DPSCs had therapeutic effects on diabetic polyneuropathy through its angiogenic and anti‐inflammatory effects. As a result, the immunosuppressed function of DPSCs could play a key role in repair and regeneration of oviduct injuries.

Growth factors such as VEGF regulate angiogenesis, which is a crucial step in wound healing.^31^ The results of this study indicated that DPSCs could be induced to form vessel‐like structures (Figure 3), and the expression of VEGF was much higher in the DPSCs group than that in the model group (Figure 6A,B). According to some studies, DPSCs could secrete the VEGF to improve the survival of endothelial cells to promote angiogenesis.^32^, ^33^ Meanwhile, there was a study that suggested that dental pulp tissue contained a high concentration of VEGF, which provided a significant contribution to the reparative response of the pulp tissues.^34^ Furthermore, DPSCs could upregulate NG2 and play a role as the pericyte‐like cells to form vascular networks.^35^, ^36^ As a result, we concluded that DPSCs possessed the capability to promote the repair and regeneration of oviduct injuries by promoting angiogenesis.

## 5 CONCLUSION

Our results showed that DPSCs held promising potentials for the repair and regeneration of oviduct damages through dual mechanisms. DPSCs may, for starters, secrete a series of soluble small molecules to inhibit inflammation in the whole body. Secondly, DPSCs could differentiate into vascular endothelial‐like cells and promote the formation of new vessels. Taken together, DPSCs could be a promising seeding cells source for stem cell‐based systematic therapy in oviduct injuries.

Although extensive stem cell banking service currently exists around the world, its clinical application is subject to technical and regulatory restrictions. The paracrine mechanisms of stem cells have been increasingly explored, including the production of extracellular vesicles, cytokines and chemokines. These small molecules may not only replace stem cells in light of their powerful therapeutic roles but also avoid the typical problems associated with cell therapy.^24^

On the other hand, single application of stem cells or small molecules secreted from stem cells may not provide the best therapeutic effect on the oviduct injuries. With the development of biomaterials, some smart materials such as hydrogel,^37^ mesoporous silica nanoparticles,^38^ could be used as the drug delivery systems to encapsulate stem cells or small molecules, in order to maintain its biological activities and ensure its sufficient amount in injured sites. Therefore, the spatiotemporal combination of stem cells or small molecules, and smart materials would provide a promising strategy for treating oviduct injuries in future clinical approaches.

## AUTHOR CONTRIBUTIONS

Qingsong Ye and Lihua Luo conceived this study; Qingsong Ye, Yilong Ai and Yan He designed the methods; Lihua Luo and Zhenjie Xing performed the majority of experiments and analysed the data; Xiangyan Liao collected the data and performed the statistical analysis; Yejian Li cultured DPSCs; Yu Luo performed the multi‐differentiation of DPSCs; Qingsong Ye, Yilong Ai and Yan He designed and coordinated the experimental procedures; Lihua Luo and Zhenjie Xing wrote the paper. All authors have read and approved the final version of the manuscript.

## CONFLICT OF INTEREST

The authors declare that they have no competing interests regarding the publication of this paper.

## Data Availability

The datasets for this study are available on request to the corresponding authors.

## References

[1] Lindsay TJ , Vitrikas KR . Evaluation and treatment of infertility. Am Fam Physician. 2015;91(5):308‐314. 25822387

[2] Lyons RA , Saridogan E , Djahanbakhch O . The reproductive significance of human fallopian tube cilia. Hum Reprod Update. 2006;12(4):363‐372. 16565155 10.1093/humupd/dml012

[3] Luo HJ , Xiao XM , Zhou J , Wei W . Therapeutic influence of intraperitoneal injection of Wharton's jelly‐derived mesenchymal stem cells on oviduct function and fertility in rats with acute and chronic salpingitis. Genet Mol Res. 2015;14(2):3606‐3617. 25966129 10.4238/2015.April.17.10

[4] Yamada Y , Nakamura‐Yamada S , Kusano K , Baba S . Clinical potential and current progress of dental pulp stem cells for various systemic diseases in regenerative medicine: a concise review. Int J Mol Sci. 2019;20(5):1132. 10.3390/ijms20051132 PMC6429131 30845639

[5] Tao X , Sun M , Chen M , et al. HMGB1‐modified mesenchymal stem cells attenuate radiation‐induced vascular injury possibly via their high motility and facilitation of endothelial differentiation. Stem Cell Res Ther. 2019;10(1):92. 30867070 10.1186/s13287-019-1197-x PMC6416980

[6] Dilger N , Neehus AL , Grieger K , Hoffmann A , Menssen M , Ngezahayo A . Gap junction dependent cell communication is modulated during transdifferentiation of mesenchymal stem/stromal cells towards neuron‐like cells. Front Cell Dev Biol. 2020;8:869. 32984345 10.3389/fcell.2020.00869 PMC7487424

[7] Ding Y , Johnson R , Sharma S , Ding X , Bryant SJ , Tan W . Tethering transforming growth factor β1 to soft hydrogels guides vascular smooth muscle commitment from human mesenchymal stem cells. Acta Biomater. 2020;105:68‐77. 31982589 10.1016/j.actbio.2020.01.034 PMC7339826

[8] Han Y , Li X , Zhang Y , Han Y , Chang F , Ding J . Mesenchymal stem cells for regenerative medicine. Cell. 2019;8(8):886. 10.3390/cells8080886 PMC6721852 31412678

[9] Jiang W , Xu J . Immune modulation by mesenchymal stem cells. Cell Prolif. 2020;53(1):e12712. 31730279 10.1111/cpr.12712 PMC6985662

[10] Gardin C , Bosco G , Ferroni L , et al. Hyperbaric oxygen therapy improves the osteogenic and vasculogenic properties of mesenchymal stem cells in the presence of inflammation in vitro. Int J Mol Sci. 2020;21(4):1452. 10.3390/ijms21041452 PMC7073059 32093391

[11] Tsutsui TW . Dental pulp stem cells: advances to applications. Stem Cells Cloning. 2020;13:33‐42. 32104005 10.2147/SCCAA.S166759 PMC7025818

[12] Staniowski T , Zawadzka‐Knefel A , Skośkiewicz‐Malinowska K . Therapeutic potential of dental pulp stem cells according to different transplant types. Molecules. 2021;26(24):7423. 34946506 10.3390/molecules26247423 PMC8707085

[13] Zou T , Jiang S , Dissanayaka WL , et al. Sema4D/PlexinB1 promotes endothelial differentiation of dental pulp stem cells via activation of AKT and ERK1/2 signaling. J Cell Biochem. 2019;120(8):13614‐13624. 30937968 10.1002/jcb.28635

[14] Gong T , Xu J , Heng B , et al. EphrinB2/EphB4 signaling regulates DPSCs to induce sprouting angiogenesis of endothelial cells. J Dent Res. 2019;98(7):803‐812. 31017515 10.1177/0022034519843886

[15] Luzuriaga J , Irurzun J , Irastorza I , Unda F , Ibarretxe G , Pineda JR . Vasculogenesis from human dental pulp stem cells grown in matrigel with fully defined serum‐free culture media. Biomedicine. 2020;8(11):483. 10.3390/biomedicines8110483 PMC7695282 33182239

[16] Matsumura‐Kawashima M , Ogata K , Moriyama M , Murakami Y , Kawado T , Nakamura S . Secreted factors from dental pulp stem cells improve Sjögren's syndrome via regulatory T cell‐mediated immunosuppression. Stem Cell Res Ther. 2021;12(1):182. 33726818 10.1186/s13287-021-02236-6 PMC7962357

[17] Neves VCM , Yianni V , Sharpe PT . Macrophage modulation of dental pulp stem cell activity during tertiary dentinogenesis. Sci Rep. 2020;10(1):20216. 33214653 10.1038/s41598-020-77161-4 PMC7678850

[18] Omi M , Hata M , Nakamura N , et al. Transplantation of dental pulp stem cells suppressed inflammation in sciatic nerves by promoting macrophage polarization towards anti‐inflammation phenotypes and ameliorated diabetic polyneuropathy. J Diabetes Investig. 2016;7(4):485‐496. 10.1111/jdi.12452 PMC4931198 27181261

[19] Yao J , Chen N , Wang X , et al. Human supernumerary teeth‐derived apical papillary stem cells possess preferable characteristics and efficacy on hepatic fibrosis in mice. Stem Cells Int. 2020;2020:6489396. 32399047 10.1155/2020/6489396 PMC7204141

[20] Gandia C , Armiñan A , García‐Verdugo JM , et al. Human dental pulp stem cells improve left ventricular function, induce angiogenesis, and reduce infarct size in rats with acute myocardial infarction. Stem Cells. 2008;26(3):638‐645. 18079433 10.1634/stemcells.2007-0484

[21] Luo L , Albashari AA , Wang X , et al. Effects of transplanted heparin‐poloxamer hydrogel combining dental pulp stem cells and bFGF on spinal cord injury repair. Stem Cells Int. 2018;2018:2398521. 29765407 10.1155/2018/2398521 PMC5892218

[22] Luo L , He Y , Jin L , et al. Application of bioactive hydrogels combined with dental pulp stem cells for the repair of large gap peripheral nerve injuries. Bioact Mater. 2021;6(3):638‐654. 33005828 10.1016/j.bioactmat.2020.08.028 PMC7509005

[23] Patil M . Assessing tubal damage. J Hum Reprod Sci. 2009;2(1):2‐11. 19562067 10.4103/0974-1208.51335 PMC2700690

[24] Codispoti B , Marrelli M , Paduano F , Tatullo M . NANOmetric BIO‐banked MSC‐derived exosome (NANOBIOME) as a novel approach to regenerative medicine. J Clin Med. 2018;7:357. 10.3390/jcm7100357 PMC6210357 30326618

[25] Jansen RP . Endocrine response in the fallopian tube. Endocr Rev. 1984;5(4):525‐551. 6094174 10.1210/edrv-5-4-525

[26] Aggarwal R , Jain AK , Mittal P , Kohli M , Jawanjal P , Rath G . Association of pro‐ and anti‐inflammatory cytokines in preeclampsia. J Clin Lab Anal. 2019;33(4):e22834. 30666720 10.1002/jcla.22834 PMC6528584

[27] Manimtim WM , Hasday JD , Hester L , Fairchild KD , Lovchik JC , Viscardi RM . Ureaplasma urealyticum modulates endotoxin‐induced cytokine release by human monocytes derived from preterm and term newborns and adults. Infect Immun. 2001;69(6):3906‐3915. 11349058 10.1128/IAI.69.6.3906-3915.2001 PMC98421

[28] Drummond GR , Vinh A , Guzik TJ , Sobey CG . Immune mechanisms of hypertension. Nat Rev Immunol. 2019;19(8):517‐532. 30992524 10.1038/s41577-019-0160-5

[29] Andrukhov O , Behm C , Blufstein A , Rausch‐Fan X . Immunomodulatory properties of dental tissue‐derived mesenchymal stem cells: implication in disease and tissue regeneration. World J Stem Cells. 2019;11(9):604‐617. 31616538 10.4252/wjsc.v11.i9.604 PMC6789188

[30] Makino E , Nakamura N , Miyabe M , et al. Conditioned media from dental pulp stem cells improved diabetic polyneuropathy through anti‐inflammatory, neuroprotective and angiogenic actions: cell‐free regenerative medicine for diabetic polyneuropathy. J Diabetes Investig. 2019;10(5):1199‐1208. 10.1111/jdi.13045 PMC6717901 30892819

[31] Liu Y , Long L , Zhang F , et al. Microneedle‐mediated vascular endothelial growth factor delivery promotes angiogenesis and functional recovery after stroke. J Control Release. 2021;338:610‐622. 34481025 10.1016/j.jconrel.2021.08.057

[32] Janebodin K , Chavanachat R , Hays A , Reyes GM . Silencing VEGFR‐2 hampers odontoblastic differentiation of dental pulp stem cells. Front Cell Dev Biol. 2021;9:665886. 34249919 10.3389/fcell.2021.665886 PMC8267829

[33] Zhang Y , Liu J , Zou T , et al. DPSCs treated by TGF‐β1 regulate angiogenic sprouting of three‐dimensionally co‐cultured HUVECs and DPSCs through VEGF‐Ang‐Tie2 signaling. Stem Cell Res Ther. 2021;12(1):281. 33971955 10.1186/s13287-021-02349-y PMC8112067

[34] Gomez‐Sosa JF , Caviedes‐Bucheli J , Díaz Barrera LE . Gene expression of vascular endothelial growth factor a and its receptors in dental pulp of immature and mature teeth. Eur Endod J. 2021;6(3):259‐263. 34967342 10.14744/eej.2021.86580 PMC8842424

[35] Delle Monache S , Martellucci S , Clementi L , et al. In vitro conditioning determines the capacity of dental pulp stem cells to function as pericyte‐like cells. Stem Cells Dev. 2019;28(10):695‐706. 30887879 10.1089/scd.2018.0192

[36] Aksel H , Huang GT . Human and swine dental pulp stem cells form a vascularlike network after angiogenic differentiation in comparison with endothelial cells: a quantitative analysis. J Endod. 2017;43(4):588‐595. 28258811 10.1016/j.joen.2016.11.015 PMC5407702

[37] Hou Y , Deng X , Xie C . Biomaterial surface modification for underwater adhesion. Smart Mater Med. 2020;1:77‐91.

[38] Dai H , Hosseinpour S , Hua S , Xu C . Advances in porous inorganic nanomaterials for bone regeneration. Nano TransMed. 2022;1:e9130005.

